# The Effects of a Normal Rate versus a Slow Intervalled Rate of Oral Nutrient Intake and Intravenous Low Rate Macronutrient Application on Psychophysical Function – Two Pilot Studies

**DOI:** 10.3389/fpsyg.2017.01031

**Published:** 2017-06-28

**Authors:** Melanie Y. Denzer-Lippmann, Stephan Bachlechner, Jan Wielopolski, Marie Fischer, Andrea Buettner, Arndt Doerfler, Christof Schöfl, Gerald Münch, Johannes Kornhuber, Norbert Thürauf

**Affiliations:** ^1^Department of Psychiatry and Psychotherapy, Friedrich-Alexander-Universität Erlangen-NürnbergErlangen, Germany; ^2^Department of Chemistry and Pharmacy, Emil Fischer Center, Friedrich-Alexander-Universität Erlangen-NürnbergErlangen, Germany; ^3^Department of Sensory Analytics, Fraunhofer Institute for Process Engineering and Packaging IVVFreising, Germany; ^4^Department of Neuroradiology, Friedrich-Alexander-Universität Erlangen-NürnbergErlangen, Germany; ^5^Division of Endocrinology and Diabetes, Department of Medicine I, Friedrich-Alexander-Universität Erlangen-NürnbergErlangen, Germany; ^6^Department of Pharmacology, School of Medicine, University of Western Sydney, PenrithNSW, Australia

**Keywords:** food ingestion, food intake, rate of food intake, psychophysical function, food craving, parenteral food administration, parenteral food intake

## Abstract

Stomach distension and energy per time are factors influencing satiety. Moreover, different rates of nutrient intake induce different stomach distension. The goal of our studies was to elucidate the influence of different oral rates of nutrient intake (normal rate versus slow intervalled rate; study I) and intravenous low rate macronutrient application (protein, carbohydrate, fat) or placebo (study II) on psychophysical function. The pilot studies investigated the effects of 1) study I: a mixed nutrient solution (1/3 protein, 1/3 fat, 1/3 carbohydrates) 2) study II: intravenous macronutrient infusions (protein, carbohydrate, fat) or placebo on psychophysical function (mood, hunger, food craving, alertness, smell intensity ratings and hedonic ratings) in human subjects. In study I 10 male subjects (age range: 21–30 years) completed the study protocol participating in both test conditions and in study II 20 male subjects (age range: 19–41 years) completed the study protocol participating in all test conditions. Additionally, metabolic function was analyzed and cognitive and olfactory tests were conducted twice starting 100 min before the beginning of the intervention and 240 min after. Psychophysical (mood, hunger, fat-, protein-, carbohydrate-, sweets- and vegetable-craving), alertness and metabolic function tests were performed seven times on each examination day. Greater effects on hunger and food cravings were observed for normal rate of intake compared to slow intervalled rate of intake and intravenous low rate macronutrient application. Our findings potentially confirm that volume of the food ingested and a higher rate of energy per time contribute to satiety during normal rate of food intake, while slow intervalled rate of food intake and intravenous low rate macronutrient application showed no effects on satiation. Our results motivate the view that a certain amount of volume of the food ingested and a certain energy per time ratio are necessary to reduce hunger and food craving.

## Introduction

Researchers have demonstrated the importance of orosensory stimulation in combination with gastric stimulation of food ingestion on satiety (Cecil et al., 1999; Wijlens et al., 2012). In an oral and gastric manipulation study, Wijlens et al. (2012) found that only the combination of oral and gastrointestinal food application leads to decreased energy intake. Moreover, Cecil et al. (1999) demonstrated that oral administration of a high-fat meal induces a greater effect on appetite and slows gastric emptying more than a high-carbohydrate meal. This would have the effect of prolonging gastric distension. Further, Rolls et al. (1998) demonstrated that ingestion of higher volumes of isoenergetic drinks results in greater satiety compared to lower volumes. Ingestion of higher volumes leads to greater stomach distension and subsequently to greater satiety. Rolls et al. (2000) also analyzed the effect of food volume independent of energy density on satiety. The results also confirmed that ingestion of higher volumes leads to higher satiety. Generally, when food is ingested, the stomach and the small intestine expand, which can be measured by mechanosensors (Gekle et al., 2010). These sensors send information about the gastrointestinal expansion to the nucleus tractus solitarii, which inhibits the hunger center and inducing satiety (Berthoud and Neuhuber, 2000). Additionally, the eating rate could also influence food intake via differences in stomach distension, with slow eating resulting in slower gastric emptying (Robinson et al., 2014). Wijlens et al. (2016) showed that a gastric infusion of 700 kcal increased satiety and lowered subsequent food intake by 35% compared to an isovolumetric gastric infusion of 100 kcal. However, the researchers could not find any effect on food intake of eight-fold longer orosensory exposure by means of modified sham feeding. In contrast, evidence indicated that a slower eating rate is associated with lower energy intake in comparison with a faster eating rate (Robinson et al., 2014). Thus, reducing eating rate may be an effective intervention to decrease energy intake as part of behavioral strategies to prevent and treat obesity (Robinson et al., 2014). Moreover, Zhang et al. (2014) demonstrated that acute retrograde gastric electrical stimulation reduced energy intake by decreasing gastric accommodation in obese subjects. Furthermore, Mion et al. (2005) observed in obese subjects that gastric emptying rates and plasma ghrelin levels were decreased in the presence of an intragastric balloon. The researchers also reported that the presence of the balloon in the stomach was associated with a significant decrease in ghrelin secretion, despite the concomitant weight loss. A pure mechanical gastric distension study performed by Wang et al. (2008) provided evidence that the left amygdala and insula process interoceptive signals of fullness produced by gastric distention involved in the control of food intake. However, Oesch et al. (2006) observed that transient pure mechanical distension of the fundus or the antrum prior to a meal does not trigger satiation. Differences in physiological and non-physiological gastric distension were observed in a H_2_^15^O-PET study (Geeraerts et al., 2011). The investigators found different regional brain activity during physiological gastric distension compared to balloon distension and interpreted the results as a prerequisite for tolerance of normal meal volumes. All these findings demonstrate that physiological gastric distension is clearly more complex than non-physiological balloon distension (Coen, 2011).

Food intake also influences human olfaction. For example, Ramaekers et al. (2016) found that participants had a higher olfactory sensitivity in the hunger state than in the satiated state. However, the researchers could not find a sensory-specific satiety. Many researchers observed changes in olfactory detection thresholds depending on food intake (Guild, 1956; Furchtgott and Friedman, 1960; Berg et al., 1963; Albrecht et al., 2009; Stafford and Welbeck, 2011). However, the results of these studies were inconsistent. In our studies, we also addressed this topic and monitored olfactory and cognitive functioning.

Psychophysical parameters are also affected by food intake. Macht et al. (2003) demonstrated that, with increasing energy density of food, negative emotions are induced directly after intake. Keith et al. (1991) observed that the mood decreased after low carbohydrate food diet for 1 week compared to moderate and high carbohydrate diet. Moreover, many studies showed that fat ingestion has a relatively weak impact on satiety, thus a high fat diet leads to weight gain because more food has to be consumed to feel satiated (de Castro, 1987; Lissner et al., 1987; Bellisle et al., 1998). In addition, protein consumption suppresses subsequent food consumption and has a higher satiating effect than carbohydrate and fat (de Castro, 1987; Poppitt et al., 1998).

Furthermore, in order to investigate the effects of different rates of nutrient intake on hunger and satiation motivating consumption, Stratton et al. (2003, 2008) conducted two separate studies employing the nasogastric tube feeding technique. It could be demonstrated that short-term continuous tube feeding could not suppress appetite and food intake (Stratton et al., 2003), whereas bolus tube feeding could suppress food intake (Stratton et al., 2008). The effects of normal oral intake versus slow intervalled oral intake on psychophysical function still remain to be investigated. We hypothesize that – in contrast to a normal rate of oral food intake – a slow intervalled rate of food intake and intravenous low rate macronutrient application will not change the perception of satiation and hunger because of the lack of gastric distension and/or the low energy per time ratio. In order to test the hypothesis, we conducted an oral study investigating the influence of normal eating rate versus a slow intervalled rate (study I) and a parenteral study investigating the influence of type of intravenous macronutrient infusion (study II) on satiety and hunger, and we analyzed additional effects on psychophysical, metabolic, olfactory, and cognitive functions.

## Materials and Methods

### Study I

#### Participants

Ten healthy young male volunteers with a mean BMI of 23.14 ± 1.64 kg/m^2^ (SD) participated in this study [age range: 21–30 years, mean age: 24.50 ± 3.72 years (SD)].

Exclusion criteria consisted of severe psychiatric illness, judged by structured clinical interview for DSM-IV and Beck-Depressions-Inventory, vegan lifestyle or unusual eating habits (FEV-questionnaire regarding eating behavior; this questionnaire is used to check for normal eating behavior. It asks for symptoms of binge eating and other eating disorders. We used this questionnaire to ensure that our subjects did not suffer from eating disorders.). Further exclusion criteria were somatic illness and abnormal hemogram, drug use, known intolerance or allergic reaction to substances contained in the nutrient solutions, smoking, BMI > 25 kg/m^2^, age under 18 and over 45 years, and severe olfactory dysfunction assessed by olfactory and gustatory clinical history and olfactory testing, i.e., inclusion in the study requested that *n*-butanol (highest concentration) and all odors of the identification test were judged to be clearly perceived.

All volunteers fulfilling none of these criteria were included. Volunteers were recruited via the homepage of the university clinical center and via bulletins on community boards at the Friedrich-Alexander-Universität Erlangen-Nürnberg. All experimental procedures were clearly explained, and volunteers provided written informed consent prior to the testing sessions. Participants were free to interrupt the testing sessions at any time. This study was carried out in accordance with the recommendations of the Declaration of Helsinki with written informed consent from all subjects. All subjects gave written informed consent in accordance with the Declaration of Helsinki. The protocol was approved by the Ethics Committee of the Friedrich-Alexander-Universität Erlangen-Nürnberg.

#### Design

**FIGURE 1:**
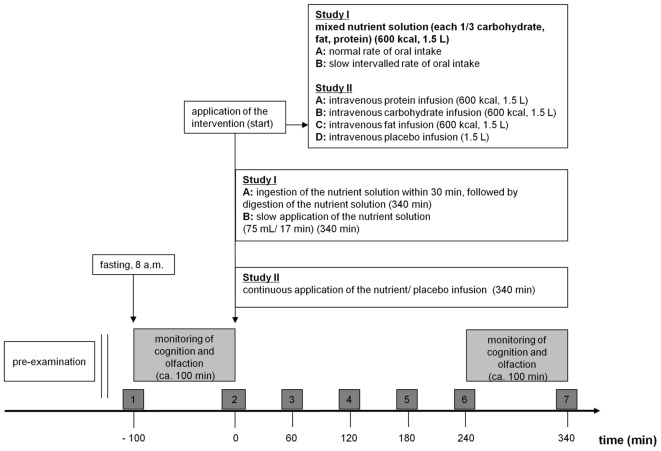
Study design (study I and II). 08:00 start of the examination day. Dark gray boxes (1–7): Registering of alertness and psychophysical parameters (mood, hunger, fat-craving, protein-craving, carbohydrate-craving, sweets-craving, and vegetable-craving, alertness) via VAS and collecting of blood samples for measuring metabolic parameters (insulin, glucose, triglycerides, urea). Bright gray boxes: These two test sessions took about 100 min and included a cognitive computer test (alertness, working memory, incompatibility) and an olfactory test [*n*-butanol threshold, discrimination, identification and intensity and hedonic evaluation (part of psychophysical test)] and were performed in pre-intake/ -infusion status and in post-intake/ on-infusion status. Study I: Participants ingested the nutrient solution depending on examination day at (A) normal rate of oral intake or (B) slow intervalled rate of oral intake. Study II: Participants received the intravenous infusions depending on examination day (A) protein or (B) carbohydrate or (C) fat or (D) placebo.

A randomized, cross-over, repeated measurement design was employed for the study. The study consisted of two testing days with a 4-16 inter-day period. On the different testing days, participants consumed a mixed nutrient solution (1/3 fat, 1/3 protein, 1/3 carbohydrates) at a normal rate or a slow intervalled rate of oral nutrient intake. The application order was randomized, i.e., 50% of the participants started with a normal rate and 50% started with a slow intervalled rate of oral nutrient intake. **Figure 1** shows the study design, including all test sessions and all parameters tested. Cognitive and olfactory testing were executed twice starting 100 min before intake of the nutrient solution (pre-intake status following overnight fasting) and 240 min after the beginning of intake of the nutrient solutions (post-intake status).

#### Nutrient Solutions

Two isovolumetric (1500 mL) and isoenergetic (600 kcal) nutrient solutions were administered. The nutrient solution consisted of inulin (1.50 g; Spinnrad GmbH, Segeberg), carboxymethyl cellulose (3.25 g, Dagmar Köhler, Alpen), glucose (21.33 g; Sigma Aldrich Chemie GmbH, Steinheim), maltodextrin (30.91 g; Sigma Aldrich Chemie GmbH, Steinheim), whey protein (52.61 g; Iron Maxx Sporternährung, Köln), lecithin (5.00 g; Spinnrad GmbH, Segeberg), aspartame (0.15 g; Acros Organics, Belgium), Liquigen (36.53 mL; Nutricia GmbH, Erlangen), food dye white (10 mL; pati-Versand GmbH, Herzlake), caramel flavor (5.00 mL; Dagmar Köhler, Alpen), and was dissolved in water (Evian, Danone Waters Deutschland GmbH) to achieve a volume of 1500 mL. The drinks were administered in opaque cups, covered by lids. Nutrient solutions were freshly prepared in the kitchen and stored in the refrigerator until consumption.

Normal rate of oral intake: The intake of the nutrient solution was established by drinking a volume of 1500 mL (evenly distributed with 8 cups) within 30 min (time per cup: 225 s, volume per cup: 187.5 mL).

Slow intervalled rate of oral intake: Intake of 75 mL of the liquid solution within 1 min every 17 min was established by drinking the identical volume distributed over the entire time period of 340 min (75mL/ 17 min/340 min). We used a timer to control for identical time intervals between solution intakes.

#### Psychophysical Function

All psychophysical functions (mood, hunger, food craving, alertness, intensity and hedonic ratings of odor pens) were registered by visual analog scales. Mood, hunger, food craving and alertness were tested shortly before blood samples were collected from the participants. Subjects rated ‘hunger,’ and ‘food craving’ employing visual analog scales (ranging from -10, to 10, including 0 as a neutral point; hunger: -10 (maximal satiety), 10 (maximal hunger); food craving: -10 (no craving), 10 (maximal craving). Food cravings were rated following the presentation of five pictures [order of pictures: (1) fat-rich food, (2) protein-rich food, (3) carbohydrate-rich food, (4) sweets, (5) vegetable]. Each visual presentation lasted 5 s. Mood was rated using the Kunin scale (Kunin, 1955). This is an ordinal scale and measures the non-numeric concept of happiness employing seven different faces expressing the status ‘very happy,’ ‘happy,’ ‘little happy,’ ‘neutral,’ ‘little sad,’ ‘sad,’ ‘very sad,’ Subjects had to choose one of the seven faces to describe their current mood.

Intensity and hedonic (unpleasantness/ pleasantness) ratings of odors: Subsequent to the identification of an odor subjects rated the intensity (20 cm scale, 0 very low intensity, 20 very high intensity) and the pleasantness (-10 to 10 cm scale, -10 very unpleasant, 10 very pleasant) of the odor employing an analog rating scale. Hedonic and intensity ratings were registered at the end of the cognitive and olfactory testing session 1 and 2, i.e., (1) directly before ingestion of the nutrient solution and (2) directly before the end of the observation ( = 340 min after initial application of the nutrient solution, see **Figure 1**) period.

#### Metabolic Function

Directly after arriving, participants received a continuous i.v. line for blood sample collection. Only small amounts of the blood plasma and serum were used for the analyses. The analyses were conducted in the central laboratory of the university hospital.

Insulin: Blood samples were collected in tubes (Sarstedt AG & Co.KG, Nümbrecht, Germany) that contained coagulation activators. The blood serum insulin level was determined by chemiluminescent immunoassay technology using LIAISON Insulin (DiaSorin Deutschland GmbH, Dietzenbach).

Glucose: Blood samples were collected in tubes (Sarstedt AG & Co.KG, Nümbrecht, Germany) that contained NaF (1.0 mg/mL blood) and EDTA (1.2 mg/mL blood). Blood plasma glucose level was determined by photometric measurement techniques via hexokinase method using AU5800 Clinical Chemistry System (Beckman Coulter GmbH, Germany, Krefeld).

Triglyceride: Blood samples were collected in tubes (Sarstedt AG & Co.KG, Nümbrecht, Germany) that contained coagulation activators. Blood serum triglyceride level was determined by photometric measurement techniques via the colorimetric method using AU5800 Clinical Chemistry System (Beckman Coulter GmbH, Germany, Krefeld).

Urea: Blood samples were collected in tubes (Sarstedt AG & Co.KG, Nümbrecht, Germany) that contained coagulation activators. Blood serum urea level was determined by photometric measurement techniques via the kinetic measurement of urease using AU5800 Clinical Chemistry System (Beckman Coulter GmbH, Germany, Krefeld).

#### Cognitive Function

All cognitive tests were performed on a computer using the Tests for Attentional Performance 2.2 (Vera Fimm, Herzogenrath, Germany).

Alertness (with and without warning tone), working memory (advanced version) and incompatibility were tested according to the instruction manual (VeraFimm, 2016). For the experimental determination of alertness, the reaction time was analyzed under two conditions. The first concerned simple reaction time measurements, in which a cross appeared on the monitor at randomly varying intervals. Upon seeing the cross, the participants were to respond as quickly as possible by pressing a key (intrinsic alertness). The second condition consisted of measuring the reaction time in response to a critical stimulus preceded by a cue stimulus presented as a warning tone. Incompatibility appears in a conflict situation in that divergent stimulus information has to be processed in parallel, thus triggering different reaction tendencies. Within the used test, arrows, which were directed to the left or the right were presented on the left or the right of a fixation point. Depending on the direction of the arrow, the participant should respond with the right or left hand irrespective of the side on which the arrow was presented.

#### Olfactory Function

For olfactory testing (threshold for *n*-butanol, discrimination, identification), the validated Sniffin’ Sticks test battery (Burghart Messtechnik GmbH, Wedel, Germany) was used (Hummel et al., 1997; Kobal et al., 2000; Denzer et al., 2014). The threshold and discrimination test sets consist of 16 triplet pen sets each. Each triplet of the threshold test contained one target odor pen and two blanks, whereas each triplet of the discrimination test contained two pens with the same odor and a third one with a different odor. The pens of each triplet were presented in random order. During the test, the examiner wore odorless gloves.

For the threshold test, we used a single up-down staircase method (Hummel et al., 1997). In addition the 16 pens of the identification test were consecutively evaluated (see psychophysical Function).

#### Statistical Analyses

Data were analyzed using SPSS (version 22.0 for Windows, SPSS IBM). We tested for normal distribution employing the Shapiro Wilk test. Mauchly’s test was used to measure sphericity. If sphericity was violated, Greenhouse–Geisser corrections were applied. To compare olfaction and cognition in pre-intake and post-intake status depending on the rate of nutrient intake, and to compare each of the seven measurement points of alertness and the psychophysical and metabolic parameters depending on the rate of nutrient intake, our data were subjected to a two-way repeated-measurement analysis of variance (ANOVA) with ‘time’ and ‘rate of nutrient intake’ as within-subject factors. The Bonferroni test was used for *post hoc* testing. In the case of non-normal distribution, non-parametric testing was executed employing the Friedman test and the Wilcoxon *post hoc* test.

Delta (post-intake minus pre-intake) of the olfactory parameters: To compare the delta of both application forms a paired *t*-test was performed. In the case of non-normal distribution, non-parametric testing was executed employing the Wilcoxon test.

Base-to-Peak analyses of metabolic parameters: To compare each measurement point of the metabolic parameters (post-intake to pre-intake; base = sessions 2, 0 min), we employed paired *t*-tests for both conditions separately (normal rate of oral intake and slow intervalled rate of oral intake).

### Study II

#### Participants

Twenty healthy young male volunteers with a mean BMI of 23.77 ± 1.73 kg/m^2^ (SD) participated in this study [age range: 19–41 years, mean age: 24.30 ± 4.70 years (SD)]. None of the participants of study I were included in study II.

Information about exclusion criteria, recruitment, participants’ consent and ethics principals is provided in Section “Participants”.

#### Design

A randomized, cross-over, repeated measurement design was employed for the study. The study consisted of four testing days with a 3–14 inter-day period. During each testing day, participants received different intravenous nutrient infusions (protein, carbohydrates, fat) or placebo within 340 min. The application order was randomized, i.e., that each 25% of the panelists started with an intravenous protein, carbohydrate, fat or placebo infusion. **Figure 1** shows the study design, including all test sessions and all parameters tested. Cognitive and olfactory testing were executed twice starting 100 min before application of the intravenous infusion (pre-infusion status following overnight fasting) and 240 min after the beginning of application of the intravenous infusion (on-infusion status).

#### Intravenous Infusions

Four isovolumetric (1500 mL) and isoenergetic (600 kcal) intravenous nutrient infusions and placebo (1500 mL) were administered (protein: Aminoplasmal B. Braun 10%-Infusionslösung, B. Braun Melsungen AG, Melsungen and Glucose 10% m/v Infusionslösung, B. Braun Melsungen AG, Melsungen; carbohydrate: Glucose 10%m/v Infusionslösung, B. Braun Melsungen AG, Melsungen; fat: Lipofundin 10% mit MCT-Infusionsflasche, B. Braun Melsungen AG, Melsungen; placebo: Kochsalz “Braun” 0,9%-Infusionslösung, B. Braun Melsungen AG, Melsungen). The intravenous infusions were continuously administered within 300 min.

#### Psychophysical Function

Information is provided in Section “Psychophysical Function”.

#### Metabolic Function

Information is provided in Section “Metabolic Function”.

#### Cognitive Function

Information is provided in Section “Cognitive Function”.

#### Olfactory Function

Information is provided in Section “Olfactory Function”.

#### Statistical Analyses

Information about software, normal distribution and sphericity is provided in Section “Statistical Analyses”.

Comparison of olfaction and cognition in pre-infusion and on-infusion status depending on the type of intravenous infusion, and comparison each of the seven measurement points of alertness, the psychophysical and metabolic parameters depending on the type of intravenous infusion and base to peak analyses of metabolic parameters were conducted analogous to study I (see Statistical Analyses).

For comparison of the different intravenous infusions at each measurement point (1–7 for psychophysical and metabolic factors and 1–2 for olfactory factors), we used a one-way repeated-measurement ANCOVA with ‘infusion’ as within-subject factor. The Bonferroni test was used for *post hoc* testing. In the case of non-normal distribution, non-parametric testing was executed employing the Friedman test and the Wilcoxon *post hoc* test.

Delta (post-intake minus pre-intake) of the olfactory parameters: To compare the delta of the four intravenous infusions a one-way repeated-measurement ANCOVA with ‘infusion’ as within-subject factor was performed. In the case of non-normal distribution, non-parametric testing was executed employing the Friedman test and the Wilcoxon *post hoc* test.

## Results

### Study I

#### Psychophysical Function

Mood: The factors ‘time,’ ‘rate of intake,’ and ‘time x rate of intake’ had no significant impact on mood [‘time’: *F*(1,6) = 0.06, *p* = 0.82; ‘rate of intake’: *F*(1,1) = 2.8, *p* = 0.052; ‘time x rate of intake’: *F*(1,6) = 2.3, *p* = 0.078].

**FIGURE 2:**
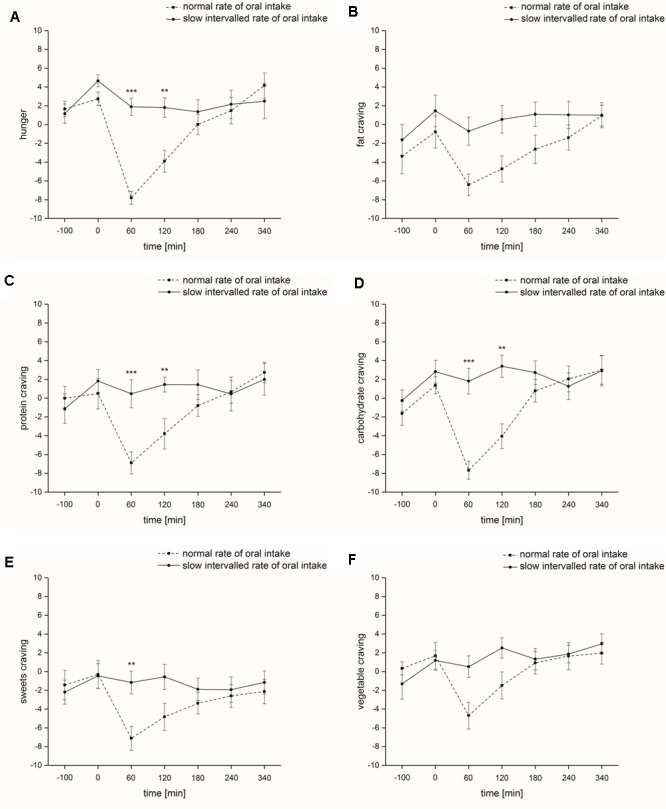
Psychophysical parameters (study I). Time course and standard errors of means of the mean psychophysical parameters **(A)** hunger, **(B)** fat craving, **(C)** protein craving, **(D)** carbohydrate craving, **(E)** sweets craving, and **(F)** vegetable craving of all participants (*n* = 10) during normal rate of oral intake and slow intervalled rate of oral intake of the nutrient solution [^∗∗^indicates a statistical difference (*p* ≤ 0.01) between both conditions at the respective time; ^∗∗∗^indicates a statistical difference (*p* ≤ 0.001) between both conditions at the respective time].

Hunger: The factors ‘time’ and ‘rate of intake’ had a significant impact on hunger [‘time’: *F*(1,6) = 13.4, *p* ≤ 0.001; ‘rate of intake’: *F*(1,1) = 9.7, *p* ≤ 0.001]. The normal rate of nutrient intake also had an influence on hunger state, i.e., we found a significant effect for ‘time x rate of intake’ [*F*(1,6) = 11.2, *p* ≤ 0.001]. At testing sessions 3 (60 min after the start of nutrient intake, *p* ≤ 0.001) and 4 (120 min after the start of nutrient intake, *p* ≤ 0.01) during normal rate of nutrient intake, participants rated their individual hunger significantly lower than during slow intervalled rate of intake, i.e., after normal rate of nutrient intake we noticed a sharp and significant drop followed by a gradual rise of hunger ratings, whereas the ratings did not change after slow intervalled rate of intake (**Figure 2A**).

Food craving: Food craving for different macronutrients was significantly affected by the factor ‘time’ [fat-rich food: *F*(1,6) = 8.8, *p* ≤ 0.001; protein-rich food: *F*(1,6) = 8.9, *p* ≤ 0.001; carbohydrate-rich food: *F*(1,6) = 12.4, *p* ≤ 0.001; sweets: *F*(1,6) = 3.9, *p* ≤ 0.05; vegetable: *F*(1,6) = 7.0, *p* ≤ 0.001]. ‘Rate of intake’ significantly affected fat, carbohydrate and sweets craving [fat-rich food: *F*(1,1) = 6.7, *p* ≤ 0.05; carbohydrate-rich food: *F*(1,1) = 9.8, *p* ≤ 0.05; sweets: *F*(1,1) = 8.6, *p* ≤ 0.05], while protein and vegetable craving was not affected [protein-rich food: *F*(1,1) = 2.9, *p* = 0.12; vegetable: *F*(1,1) = 3.7, *p* = 0.085]. ‘Time x rate of intake’ interaction also significantly influenced protein, carbohydrate and sweets craving [protein-rich food: *F*(1,6) = 6.1, *p* ≤ 0.01; carbohydrate-rich food: *F*(1,6) = 6.5, *p* ≤ 0.01; sweets: *F*(1,6) = 4.3, *p* ≤ 0.05], while fat and vegetable craving was not affected [fat-rich food: *F*(1,6) = 2.3, *p* = 0.11; vegetable: *F*(1,6) = 2.7, *p* = 0.075]. At testing sessions 3 (60 min after the start of nutrient intake, protein-rich food: *p* ≤ 0.001; carbohydrate-rich food: *p* ≤ 0.001; sweets: *p* ≤ 0.01) and 4 (120 min after the start of nutrient intake, protein-rich food: *p* ≤ 0.01; carbohydrate-rich food: *p* ≤ 0.01) following normal rate of intake, subjects rated their individual food craving significantly lower compared to slow intervalled rate of intake (**Figures 2B–F**). Alertness: The factors ‘time,’ ‘rate of intake’ and ‘time x rate of intake’ had no significant impact on alertness [‘time’: *F*(1,6) = 1.3, *p* = 0.30; ‘rate of intake’: *F*(1,1) = 0.89, *p* = 0.37; ‘time x rate of intake’: *F*(1,6) = 0.97, *p* = 0.43].

Intensity ratings of odors: We found no significant effect of the factor ‘rate of intake’ and ‘time x rate of intake’ on intensity ratings [‘rate of intake’: *F*(1,1) = 2.7, *p* = 0.14; ‘time x rate of intake’: *F*(1,1) = 0.40, *p* = 0.54]. However, ‘time’ significantly affected intensity ratings [*F*(1,1) = 5.6, *p* ≤ 0.05], i.e., the odors were perceived more intensely in the post-intake status compared to pre-intake status (normal rate of intake: pre-intake status: 12.7 ± 1.2, post-intake status: 13.5 ± 1.6; slow intervalled rate of intake: pre-intake status: 13.5 ± 1.8, post-intake status: 13.8 ± 1.5). This was independent of intake rate.

Hedonic ratings of odors: We found no significant effect of the factor ‘time’ on hedonic ratings [*F*(1,1) = 2.3, *p* = 0.17]. However, ‘rate of intake’ and ‘time x rate of intake’ significantly affected hedonic ratings (‘rate of intake’: [*F*(1,1) = 7.7, *p* ≤ 0.05; ‘time x rate of intake’: *F*(1,1) = 8.0, *p* ≤ 0.05]. *Post hoc* analysis demonstrated a significant difference at test session 1 (*p* ≤ 0.01), i.e., participants rated the odorants more pleasant in the slow intervalled intake setting compared to the normal intake setting (normal rate of intake: pre-intake status: 0.83 ± 1.7, post-intake status: 0.88 ± 1.9; slow intervalled rate of intake: pre-intake status: 1.8 ± 1.9, post-intake status: 1.2 ± 2.0).

#### Metabolic Function

##### Insulin

**FIGURE 3:**
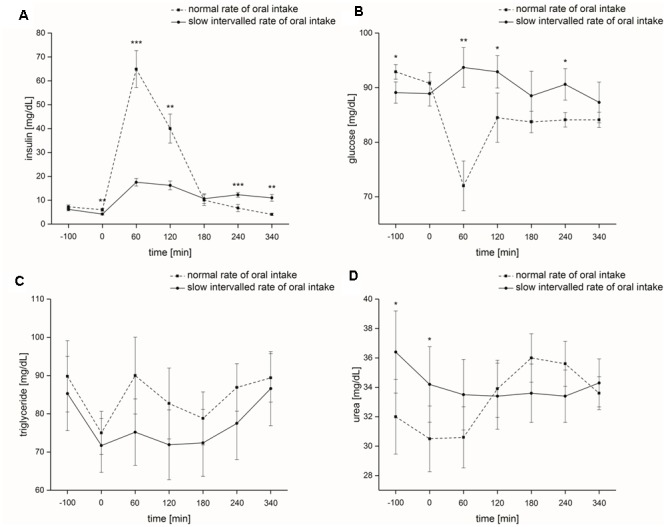
Metabolic parameters (study I). Time course and standard errors of means of the mean metabolic parameters **(A)** insulin, **(B)** glucose, **(C)** triglyceride, and **(D)** urea of all participants (*n* = 10) during normal rate of oral intake and slow intervalled rate of oral intake of the nutrient solution [^∗^indicates a statistical difference (*p* ≤ 0.05) between both conditions at the respective time; ^∗∗^indicates a statistical difference (*p* ≤ 0.01) between both conditions at the respective time; ^∗∗∗^indicates a statistical difference (*p* ≤ 0.001) between both conditions at the respective time].

The factors ‘time’ and ‘rate of intake’ had a significant impact on blood serum insulin levels [‘time’: *F*(1,6) = 41.7, *p* ≤ 0.001; ‘rate of intake’: *F*(1,1) = 24.9, *p* ≤ 0.001]. ‘Time x rate of intake’ interaction also significantly influenced blood serum insulin levels [*F*(1,6) = 36.0, *p* ≤ 0.001]. At testing sessions 2 (0 min directly before the start of nutrient intake, *p* ≤ 0.01), 3 (60 min after the start of nutrient intake, *p* ≤ 0.001) and 4 (120 min after the start of nutrient intake, *p* ≤ 0.05) blood serum insulin levels were significantly higher following normal rate of intake and at testing sessions 6 (240 min after the start of nutrient intake, *p* ≤ 0.001) and 7 (340 min after the start of nutrient intake, *p* ≤ 0.01) blood serum insulin levels were significantly higher following slow intervalled rate of intake (**Figure 3A**). Differences in base-to-peaks ratios (testing session 2 compared to testing sessions 3–7) for normal rate of intake are presented in **Table 1**.

**Table 1:** Numerical difference of means of the metabolic parameters (study I and II).

	Insulin		Glucose		Triglyceride		Urea	
	Difference [mg/dL]	*p*-value	Difference [mg/dL]	*p*-value	Difference [mg/dL]	*p*-value	Difference [mg/dL]	*p*-value
**Study I**								
Normal rate of oral intake								
m3-m2	58.9	0.001	18.8	0.001	15.0	0.05	0.01	0.76
m4-m2	34.0	0.001	6.3	0.19	7.7	0.18	3.4	0.001
m5-m2	4.0	0.08	7.1	0.05	3.1	0.44	5.5	0.001
m6-m2	0.73	0.62	6.7	0.01	11.9	0.05	5.1	0.001
m7-m2	2.0	0.01	6.7	0.01	14.4	0.01	3.1	0.05
Slow intervalled rate of oral intake								
m3-m2	13.4	0.001	4.8	0.11	3.5	0.10	0.70	0.11
m4-m2	12.0	0.001	4.0	0.06	0.2	0.95	0.80	0.10
m5-m2	6.5	0.01	0.40	0.90	0.7	0.85	0.60	0.42
m6-m2	8.1	0.001	1.7	0.58	5.8	0.29	0.80	0.42
m7-m2	6.8	0.01	1.6	0.58	14.9	0.05	0.10	0.93
**Study II**								
Protein								
m3-m2	14.6	0.001	4.4	0.06	–10.7	0.01	–0.05	0.93
m4-m2	14.0	0.001	6.7	0.01	–15.3	0.001	–0.68	0.24
m5-m2	11.5	0.001	7.4	0.001	–16.9	0.001	–0.26	0.45
m6-m2	8.0	0.001	10.1	0.054	–13.5	0.01	0	1.0
m7-m2	7.8	0.001	5.2	0.05	–10.2	0.05	–0.21	0.64
Carbohydrate								
m3-m2	17.2	0.001	40.2	0.001	–6.2	0.001	–1.8	0.001
m4-m2	17.8	0.001	25.2	0.01	–12.8	0.001	–3.4	0.001
m5-m2	12.3	0.001	16.9	0.001	–15.5	0.001	–4.5	0.001
m6-m2	11.5	0.001	26.7	0.001	–17.5	0.001	–6.0	0.001
m7-m2	8.8	0.001	12.9	0.001	–15.8	0.001	–7.7	0.001
Fat								
m3-m2	1.0	0.13	0.15	0.94	95.3	0.001	–1.9	0.001
m4-m2	0.02	0.98	0.95	0.41	145.0	0.001	–2.8	0.001
m5-m2	0.53	0.47	0.80	0.58	157.7	0.001	–3.6	0.001
m6-m2	0.20	0.76	–1.5	0.35	168.0	0.001	–5.0	0.001
m7-m2	–0.20	0.82	–3.9	0.05	166.2	0.001	–6.5	0.001
Placebo								
m3-m2	–0.70	0.24	–1.2	0.45	–5.5	0.05	–1.5	0.05
m4-m2	–1.1	0.09	–1.9	0.14	–9.0	0.05	–1.5	0.001
m5-m2	–1.8	0.01	–1.7	0.23	–9.7	0.05	–2.6	0.001
m6-m2	–2.2	0.01	–3.4	0.05	–10.9	0.05	–3.3	0.001
m7-m2	–2.1	0.09	–2.8	0.11	–12.0	0.01	–4.6	0.001

*m, mean of session.*

##### Glucose

Blood plasma glucose levels were not significantly affected by the factor ‘time’ [*F*(1,6) = 2.5, *p* = 0.079]. ‘Rate of intake’ had a significant effect on blood plasma glucose levels [*F*(1,1) = 8.7, *p* ≤ 0.05]. We also found a significant effect for ‘time x rate of intake’ on blood plasma glucose levels [*F*(1,6) = 9.1, *p* ≤ 0.001]. After start of intake of the nutrient solution, blood plasma glucose levels were significantly higher following the slow intervalled rate of intake at testing sessions 3 (60 min after the start of nutrient intake, *p* ≤ 0.001), 4 (120 min after the start of nutrient intake, *p* ≤ 0.05) and 6 (240 min after the start of nutrient intake, *p* ≤ 0.05) (**Figure 3B**). Differences base-to-peak ratios (testing session 2 compared to testing sessions 3–7) for normal rate of intake are presented in **Table 1**.

##### Triglycerides

The factors ‘time,’ ‘rate of intake,’ and ‘time x rate of intake’ had no significant impact on blood serum triglyceride levels [‘time’: *F*(1,6) = 2.8, *p* = 0.10; ‘rate of intake’: *F*(1,1) = 2.6, *p* = 0.14; ‘time x rate of intake’: *F*(1,6) = 2.1, *p* = 0.14] (**Figure 3C**). Differences in base-to-peaks ratios (testing session 2 compared to testing sessions 3–7) for normal rate of intake are presented in **Table 1**.

##### Urea

The factors ‘time’ and ‘rate of intake’ did not significantly influence blood serum urea levels [‘time’: *F*(1,6) = 4.0, *p* = 0.069; ‘rate of intake’: *F*(1,6) = 0.45, *p* = 0.52]. ‘Time x rate of intake’ had a significant effect on blood serum urea levels [*F*(1,6) = 39.0, *p* ≤ 0.001]. We noticed a significant difference between the two intake conditions at base, i.e., testing sessions 1 and 2 (*p* ≤ 0.05) (**Figure 3D**). Differences base-to-peak ratios (testing session 2 compared to testing sessions 3–7) for normal rate of intake are presented in **Table 1**.

#### Cognitive Function

Alertness without acoustic signal: We found no significant effect of the factors ‘time,’ ‘rate of intake,’ and ‘time x rate of intake’ on reaction time [‘time’: *F*(1,1) = 0.77, *p* = 0.40; ‘rate of intake’: *F*(1,1) = 1.3, *p* = 0.30; ‘time x rate of intake’: *F*(1,1) = 0.51, *p* = 0.50]. The factors ‘time,’ ‘rate of intake,’ and ‘time x rate of intake’ had no significant effect on error [χ^2^(3) = 3.0, *p* = 0.39] (**Table 2**).

**Table 2:** Cognitive parameters (study I and II).

	Alertness without acoustic signal	Alertness with acoustic signal	Working memory	Incompatibility
**Study I**				
Normal rate of oral intake	Test 1: 237.7 ± 41.7	Test 1: 227.9 ± 28.0	Test 1: 649.0 ± 173.6	Test 1: 414.9 ± 76.2
RT^∗^ [ms]	Test 2: 230.4 ± 29.5	Test 2: 226.2 ± 26.7	Test 2: 554.1 ± 150.2	Test 2: 396.6 ± 58.0
Normal rate of oral intake	Test 1: 0 ± 0	Test 1: 1.3 ± 1.8	Test 1: 1.6 ± 2.3	Test 1: 1.4 ± 1.2
error	Test 2: 0.20 ± 0.63	Test 2: 1.8 ± 2.1	Test 2: 2.5 ± 2.3	Test 2: 0.7 ± 1.3
Slow intervalled rate of oral	Test 1: 225.2 ± 20.3	Test 1: 218.9 ± 26.5	Test 1: 599.7 ± 160.3	Test 1: 391.5 ± 60.3
intake RT [ms]	Test 2: 221.8 ± 22.2	Test 2: 218.5 ± 18.0	Test 2: 607.3 ± 196.4	Test 2: 392.4 ± 59.0
Slow intervalled rate of oral	Test 1: 0 ± 0	Test 1: 0.70 ± 1.3	Test 1: 2.3 ± 3.2	Test 1: 1.3 ± 1.3
intake error	Test 2: 0 ± 0	Test 2: 1.2 ± 1.3	Test 2: 2.2 ± 2.8	Test 2: 1.6 ± 1.5
**Study II**				
Protein RT [ms]	Test 1: 244.8 ± 46.6	Test 1: 237.9 ± 27.1	Test 1: 659.5 ± 159.2	Test 1: 424.6 ± 87.1
	Test 2: 241.4 ± 55.0	Test 2: 227.4 ± 24.2	Test 2: 639.1 ± 167.3	Test 2: 406.4 ± 76.5
Protein error	Test 1: 0 ± 0	Test 1: 0.58 ± 1.0	Test 1: 1.8 ± 1.4	Test 1: 1.7 ± 2.4
	Test 2: 0 ± 0	Test 2: 0.42 ± 1.2	Test 2: 1.0 ± 1.4	Test 2: 1.5 ± 1.6
Carbohydrate RT [ms]	Test 1: 243.1 ± 47.1	Test 1: 234.4 ± 26.8	Test 1: 675.6 ± 170.8	Test 1: 427.6 ± 82.2
	Test 2: 245.1 ± 50.7	Test 2: 229.8 ± 24.3	Test 2: 653.9 ± 165.4	Test 2: 418.3 ± 83.0
Carbohydrate error	Test 1: 0.05 ± 0.23	Test 1: 0.53 ± 0.90	Test 1: 0.89 ± 1.1	test 1: 1.4 ± 1.2
	Test 2: 0 ± 0	Test 2: 0.58 ± 1.3	Test 2: 0.89 ± 1.6	test 2: 1.7 ± 1.8
Fat RT [ms]	Test 1: 244.3 ± 48.9	Test 1: 238.3 ± 28.6	Test 1: 687.9 ± 171.2	Test 1: 424.6 ± 89.1
	Test 2: 241.2 ± 36.4	Test 2: 229.6 ± 27.1	Test 2: 609.7 ± 158.8	Test 2: 418.7 ± 97.9
Fat error	Test 1: 0 ± 0	Test 1: 0.37 ± 0.50	Test 1: 1.6 ± 2.1	Test 1: 1.3 ± 2.3
	Test 2: 0 ± 0	Test 2: 0.53 ± 1.4	Test 2: 1.1 ± 1.3	Test 2: 1.5 ± 1.8
Placebo RT [ms]	Test 1: 256.3 ± 73.3	Test 1: 242.5 ± 40.1	Test 1: 672.3 ± 156.6	Test 1: 404.6 ± 119.7
	Test 2: 243.0 ± 44.9	Test 2: 236.8 ± 33.3	Test 2: 643.1 ± 176.0	Test 2: 427.7 ± 98.3
Placebo error	Test 1: 0 ± 0	Test 1: 0.37 ± 0.76	Test 1: 1.2 ± 1.1	Test 1: 1.7 ± 2.2
	Test 2: 0 ± 0	Test 2: 0.68 ± 1.3	Test 2: 1.3 ± 1.2	Test 2: 1.7 ± 1.9

*^∗^RT, reaction time.*

Alertness with acoustic signal: We found no significant effect of the factors ‘time,’ ‘rate of intake,’ and ‘time x rate of intake’ on reaction time [‘time’: *F*(1,1) = 0.034, *p* = 0.86; ‘rate of intake’: *F*(1,1) = 2.5, *p* = 0.15; ‘time x rate of intake’: *F*(1,1) = 0.018, *p* = 0.90]. The factors ‘time,’ ‘rate of intake,’ and ‘time x rate of intake’ had no significant effect on error [χ^2^(3) = 3.1, *p* = 0.38] (**Table 2**).

Working memory: We found no significant effect of the factors ‘time,’ ‘rate of intake,’ and ‘time x rate of intake’ on reaction time [‘time’: *F*(1,1) = 2.2, *p* = 0.17; ‘rate of intake’: *F*(1,1) = 0.014, *p* = 0.91; ‘time x rate of intake’: *F*(1,1) = 2.3, *p* = 0.16]. The factors ‘time,’ ‘rate of intake,’ and ‘time x rate of intake’ had no significant effect on error [χ^2^(3) = 1.6, *p* = 0.66] (**Table 2**).

Incompatibility: We found no significant effect of the factors ‘time,’ ‘rate of intake,’ and ‘time x rate of intake’ on reaction time [‘time’: *F*(1,1) = 4.2, *p* = 0.072; ‘rate of intake’: *F*(1,1) = 1.1, *p* = 0.32; ‘time x rate of intake’: *F*(1,1) = 1.9, *p* = 0.20]. Error was also not significantly affected by ‘time’ and ‘rate of intake’ [‘time’: *F*(1,1) = 0.40, *p* = 0.54; ‘rate of intake’: *F*(1,1) = 1.8, *p* = 0.21]. However, our statistical analysis revealed a significant effect on error for ‘time x rate of intake’ [*F*(1,1) = 11.3, *p* ≤ 0.01]. *Post hoc* analysis demonstrated a significant difference at test session 2 (*p* ≤ 0.01), i.e., participants produced lower errors during the post-intake status following normal rate of intake (**Table 2**).

#### Olfactory Parameters

Threshold: The factors ‘time,’ ‘rate of intake,’ and ‘time x rate of intake’ had no significant influence on the *n*-butanol threshold [χ^2^(3) = 2.4, *p* = 0.49; threshold scores: normal rate of intake: pre-intake status: 9.4 ± 1.6, post-intake status: 8.7 ± 0.76; slow intervalled rate of intake: pre-intake status: 9.1 ± 1.7, post-intake status: 9.0 ± 2.2]. The delta of both application forms did not significantly differ (*p* = 0.12).

Discrimination: We found no significant effect of the factors ‘time,’ ‘rate of intake,’ and ‘time x rate of intake’ on odor discrimination [‘time’: *F*(1,1) = 0.96, *p* = 0.35; ‘rate of intake’: *F*(1,1) = 0.60, *p* = 0.46; ‘time x rate of intake’: *F*(1,1) = 4.2, *p* = 0.071], discrimination scores: normal rate of intake: pre-intake status: 12.9 ± 1.7, post-intake status: 13.3 ± 1.6; slow intervalled rate of intake: pre-intake status: 14.0 ± 1.4, post-intake status: 12.9 ± 1.8). The delta of both application forms did not significantly differ (*p* = 0.071).

**FIGURE 4:**
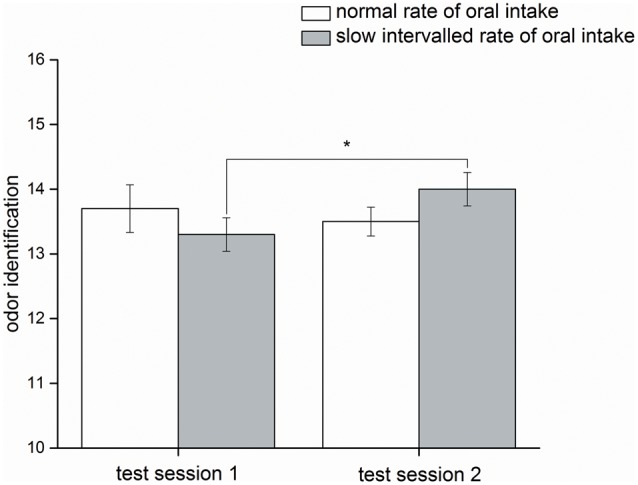
Olfactory parameter (study I). Mean values and standard errors of means of the olfactory parameter odor identification for all participants (*n* = 10) during normal rate of oral intake and slow intervalled rate of oral intake of the nutrient solution. Test session 2 took place in the post-intake status and test session 1 in the pre-intake status [^∗^indicates a statistical difference (*p* ≤ 0.05) between both conditions].

Identification: Odor identification was significantly affected by the factors ‘time,’ ‘rate of intake,’ and ‘time x rate of intake’ [χ^2^(3) = 7.9, *p* ≤ 0.05]. *Post hoc* analysis demonstrated a significant difference (*p* ≤ 0.05) when comparing pre-intake and post-intake status of the slow intervalled rate of intake condition (pre-intake status: 13.3 ± 0.82, post-intake status: 14.0 ± 0.82), but this effect was not observed for the normal rate of intake condition (*p* = 0.41; pre-intake status: 13.7 ± 1.2, post-intake status: 13.5 ± 0.71) (**Figure 4**). The delta of both application forms did not significantly differ (*p* = 0.068).

### Study II

#### Psychophysical Function

Mood: ‘Time’ significantly increased mood [*F*(1,6) = 3.4, *p* ≤ 0.05]. The factors ‘infusion’ [*F*(1,3) = 0.61, *p* = 0.54] and ‘time x infusion’ [*F*(3,6) = 1.7, *p* = 0.11] had no significant impact on ‘mood.’

**FIGURE 5:**
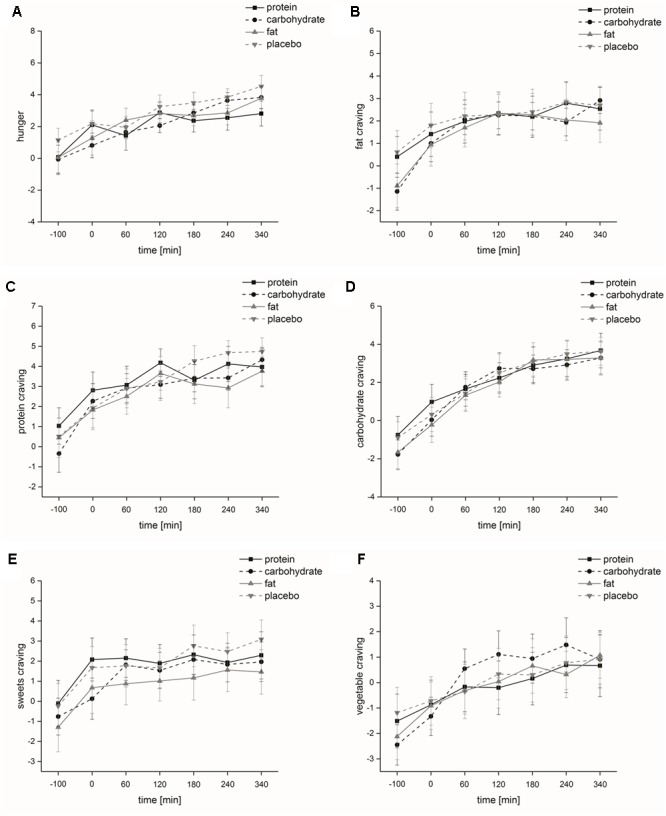
Psychophysical parameters (study II). Time course and standard errors of means of the mean psychophysical parameters **(A)** hunger, **(B)** fat craving, **(C)** protein craving, **(D)** carbohydrate craving, **(E)** sweets craving, and **(F)** vegetable craving of all participants (*n* = 20) during intravenous protein, carbohydrate, fat and placebo infusion.

Hunger: ‘Time’ significantly increased hunger [*F*(1,6) = 8.2, *p* ≤ 0.01]. The factors ‘infusion’ [*F*(1,3) = 1.4, *p* = 0.24] and ‘time x infusion’ [*F*(3,6) = 1.1, *p* = 0.38] had no significant impact on ‘hunger’ (**Figure 5A**).

Food craving: Food craving was significantly increased by the factor ‘time’ [fat-rich food: *F*(1,6) = 12.2, *p* ≤ 0.001; protein-rich food: *F*(1,6) = 16.3, *p* ≤ 0.001; carbohydrate-rich food: *F*(1,6) = 35.9, *p* ≤ 0.001; sweets: *F*(1,6) = 8.1, *p* < 0.01; vegetable: *F*(1,6) = 12.5, *p* < 0.001]. Food craving was not significantly affected by the factor ‘infusion’ [fat-rich food: *F*(1,3) = 1.2, *p* = 0.33; protein-rich food: *F*(1,3) = 1.6, *p* = 0.21; carbohydrate-rich food: *F*(1,3) = 0.46, *p* = 0.69; sweets: *F*(1,3) = 2.2, *p* = 0.12; vegetable: *F*(1,3) = 0.37, *p* = 0.72]. ‘Time x infusion’ did not significantly influence food craving [fat-rich food: *F*(3,6) = 0.91, *p* = 0.51; protein-rich food: *F*(3,6) = 1.1, *p* = 0.39; carbohydrate-rich food: *F*(3,6) = 0.57, *p* = 0.76; sweets: *F*(3,6) = 0.62, *p* = 0.70; vegetable: *F*(1,3) = 1.0, *p* = 0.41] (**Figures 5B–F**).

Alertness: The factor ‘time’ had a significant impact on alertness [*F*(1,6) = 8.7, *p* ≤ 0.001]. Alertness ratings decreased until time 3 and increased thereafter. ‘Infusion’ [*F*(1,3) = 1.5, *p* = 0.22] and ‘time x infusion’ [*F*(3,6) = 0.82, *p* = 0.59] did not significantly affect alertness.

Intensity ratings: The factors ‘time’ [*F*(1,1) = 3.2, *p* = 0.09], ‘infusion’ [*F*(1,3) = 0.97, *p* = 0.40] and ‘time x infusion’ [*F*(1,3) = 1.6, *p* = 0.22] had no significant effect on subjects’ intensity ratings.

Hedonic ratings: The factors ‘time’ [*F*(1,1) = 2.2, *p* = 0.16], ‘infusion’ [*F*(1,3) = 0.13, *p* = 0.77] and ‘time x infusion’ [*F*(1,3) = 0.91, *p* = 0.37] had no significant effect on subjects’ hedonic ratings.

#### Metabolic Function

##### Insulin

**FIGURE 6:**
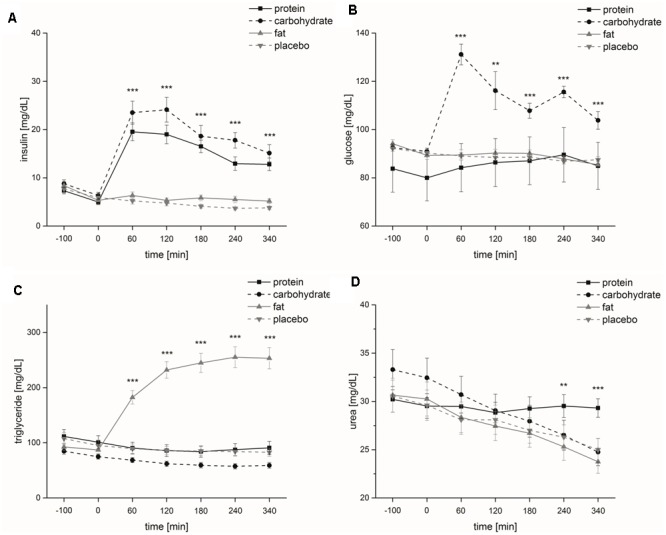
Metabolic parameters (study II). Time course and standard errors of means of the mean metabolic parameters **(A)** insulin, **(B)** glucose, **(C)** triglyceride, and **(D)** urea of all participants (*n* = 20) during intravenous protein, carbohydrate, fat and placebo infusion [^∗∗^indicates a statistical difference (*p* ≤ 0.01) between both conditions at the respective time; ^∗∗∗^indicates a statistical difference (*p* ≤ 0.001) between both conditions at the respective time].

The factors ‘time’ [*F*(1,6) = 36.3, *p* ≤ 0.001], ‘infusion’ [*F*(1,3) = 67.8, *p* ≤ 0.001] and ‘time x infusion’ [*F*(3,6) = 27.2, *p* ≤ 0.001] had a significant impact on insulin levels. *Post hoc* analyses demonstrated that insulin levels were significantly higher regarding carbohydrate intake compared to fat (*p* ≤ 0.001) and placebo (*p* ≤ 0.001) intake and significantly higher regarding protein intake compared to fat (*p* ≤ 0.001) and placebo intake (*p* ≤ 0.001). The comparison of insulin levels at each measurement point showed that at time 3 [*F*(1,3) = 66.7, *p* ≤ 0.001], time 4 [*F*(1,3) = 42.8, *p* ≤ 0.001], time 5 [*F*(1,3) = 60.9, *p* ≤ 0.001], time 6 [*F*(1,3) = 55.2, *p* ≤ 0.001] and time 7 [*F*(1,3) = 32.3, *p* ≤ 0.001] insulin levels significantly differed between the four intravenous infusions (**Figure 6A**). *Post hoc* analyses demonstrated that insulin levels were significantly higher after carbohydrate intake compared to fat (time 3: *p* ≤ 0.001; time 4: *p* ≤ 0.001; time 5: *p* ≤ 0.001; time 6: *p* ≤ 0.001; time 7: *p* ≤ 0.001) and placebo intake (time 3: *p* ≤ 0.001; time 4: *p* ≤ 0.001; time 5: *p* ≤ 0.001; time 6: *p* ≤ 0.001; time 7: *p* ≤ 0.001), insulin levels were significantly higher after protein intake compared to fat (time 3: *p* ≤ 0.001; time 4: *p* ≤ 0.001; time 5: *p* ≤ 0.001; time 6: *p* ≤ 0.001; time 7: *p* ≤ 0.001) and placebo intake (time 3: *p* ≤ 0.001; time 4: *p* ≤ 0.001; time 5: *p* ≤ 0.001; time 6: *p* ≤ 0.001; time 7: *p* ≤ 0.001) and insulin levels were significantly higher after fat intake compared to placebo intake (time 5: *p* ≤ 0.05). Differences in base-to-peak ratios (time 2 compared to time 3–7) for the four different intravenous infusions are presented in **Table 1**.

##### Glucose

The factors ‘time’ [*F*(1,6) = 8.7, *p* ≤ 0.001], ‘infusion’ [*F*(1,3) = 43.9, *p* ≤ 0.01] and ‘time x infusion’ [*F*(3,6) = 11.2, *p* ≤ 0.001] had a significant impact on glucose levels. *Post hoc* analyses demonstrated that glucose levels were significantly higher regarding carbohydrate compared to protein (*p* ≤ 0.01), fat (*p* ≤ 0.001) and placebo (*p* ≤ 0.001) intake and glucose levels were significantly higher regarding protein compared to fat (*p* ≤ 0.05) and placebo (*p* ≤ 0.01) intake. The comparison of glucose levels at each measurement point showed that at time 3 [*F*(1,3) = 63.9, *p* ≤ 0.001], time 4 [*F*(1,3) = 9.1, *p* ≤ 0.01], time 5 [*F*(1,3) = 22.5, *p* ≤ 0.001], time 6 [*F*(1,3) = 20.6, *p* ≤ 0.001] and time 7 [*F*(1,3) = 15.0, *p* ≤ 0.001] glucose levels significantly differed between the four intravenous infusions (**Figure 6B**). *Post hoc* analyses demonstrated that glucose levels were significantly higher after carbohydrate intake compared to protein (time 3: *p* ≤ 0.001; time 5: *p* ≤ 0.01), fat (time 3: *p* ≤ 0.001; time 4: *p* ≤ 0.05; time 5: *p* ≤ 0.001, time 6: *p* ≤ 0.001; time 7: *p* ≤ 0.001) and placebo intake (time 3: *p* ≤ 0.001; time 4: *p* ≤ 0.05; time 5: *p* ≤ 0.001; time 6: *p* ≤ 0.001; time 7: *p* ≤ 0.01) and glucose levels were significantly higher after protein intake compared to fat (time 5: *p* ≤ 0.01; time 7: *p* ≤ 0.01) and placebo intake (time 4: *p* ≤ 0.05; time 5: *p* ≤ 0.01; time 7: *p* ≤ 0.01). Differences in base-to-peak ratios (time 2 compared to time 3–7) for the four different intravenous infusions are presented in **Table 1**.

##### Triglycerides

The factors ‘time’ [*F*(1,6) = 18.0, *p* ≤ 0.001], ‘infusion’ [*F*(1,3) = 61.9, *p* ≤ 0.001] and ‘time x infusion’ [*F*(3,6) = 78.2, *p* ≤ 0.001] had a significant impact on triglyceride levels. *Post hoc* analyses demonstrated that triglyceride levels were significantly higher regarding fat compared to protein (*p* ≤ 0.001), carbohydrate (*p* ≤ 0.001) and placebo (*p* ≤ 0.001) intake and triglyceride levels were significantly higher regarding placebo compared to carbohydrate intake (*p* ≤ 0.05). The comparison of triglyceride levels at each measurement point showed that at time 3 [*F*(1,3) = 46.4, *p* ≤ 0.001], time 4 [*F*(1,3) = 88.2, *p* ≤ 0.001], time 5 [*F*(1,3) = 92.1, *p* ≤ 0.001], time 6 [*F*(1,3) = 91.8, *p* ≤ 0.001] and time 7 [*F*(1,3) = 86.7, *p* ≤ 0.001] triglyceride levels significantly differed between the four intravenous infusions (**Figure 6C**). *Post hoc* analyses demonstrated that triglyceride levels were significantly higher after fat intake compared to protein (time 3: *p* ≤ 0.001; time 4: *p* ≤ 0.001; time 5: *p* ≤ 0.001; time 6: *p* ≤ 0.001; time 7: *p* ≤ 0.001), carbohydrate (time 3: *p* ≤ 0.001; time 4: *p* ≤ 0.001; time 5: *p* ≤ 0.001; time 6: *p* ≤ 0.001; time 7: *p* ≤ 0.001) and placebo intake (time 3: *p* ≤ 0.001; time 4: *p* ≤ 0.001; time 5: *p* ≤ 0.001; time 6: *p* ≤ 0.001; time 7: *p* ≤ 0.001) and triglyceride levels were significantly lower after carbohydrate intake compared to protein (time 5: *p* ≤ 0.05; time 6: *p* ≤ 0.05; time 7: *p* ≤ 0.05) and placebo intake (time 4: *p* ≤ 0.05; time 5: *p* ≤ 0.05; time 6: *p* ≤ 0.01; time 7: *p* ≤ 0.01). Differences in base-to-peak ratios (time 2 compared to time 3–7) for normal rate of intake are presented in **Table 1**.

##### Urea

‘Infusion’ had no significant effect on urea levels [*F*(1,3) = 1.8, *p* = 0.18]. The factors ‘time’ [*F*(1,6) = 65.0, *p* ≤ 0.001] and ‘time x infusion’ [*F*(3,6) = 17.0, *p* ≤ 0.001] had a significant impact on urea levels. The comparison of urea levels at each measurement point showed that at time 6 [*F*(1,3) = 6.3, *p* ≤ 0.01] and time 7 [*F*(1,3) = 14.5, *p* ≤ 0.001] urea levels significantly differed between the four intravenous infusions (**Figure 6D**). *Post hoc* analyses demonstrated that urea levels were significantly higher after protein intake compared to carbohydrate (time 7: *p* ≤ 0.001), fat (time 6: *p* ≤ 0.01; time 7: *p* ≤ 0.001) and placebo (time 6: *p* ≤ 0.01; time 7: *p* ≤ 0.001) intake. Differences in base-to-peak ratios (time 2 compared to time 3–7) for the four different intravenous infusions are presented in **Table 1**.

#### Cognitive Function

Alertness without acoustic signal: We found no significant effects of the factors ‘time’ [*F*(1,1) = 3.2, *p* = 0.09], ‘infusion’ [*F*(1,3) = 1.4, *p* = 0.26] and ‘time x infusion’ [*F*(1,3) = 1.5, *p* = 0.25] on the parameter ‘alertness without acoustic signal’ on reaction time. Regarding error, the factors ‘time’ (*p* = 0.32), ‘infusion’ [χ^2^(3) = 3.0, *p* = 0.39] and ‘time x infusion’ [χ^2^(7) = 7.0, *p* = 0.43] did not affect error (**Table 2**).

Alertness with acoustic signal: In terms of reaction time, we found a significantly decreasing effect of the factor ‘time’ [*F*(1,1) = 19.1, *p* ≤ 0.001], but no significant effect of the factors ‘infusion’ [*F*(1,3) = 1.6, *p* = 0.22] and ‘time x infusion’ [*F*(1,3) = 0.35, *p* = 0.71] on the parameter ‘alertness with acoustic signal.’ Regarding error, the factors ‘time’ (*p* = 0.53), ‘infusion’ [χ^2^(3) = 0.05, *p* = 1.0] and ‘time x infusion’ [χ^2^(7) = 2.3, *p* = 0.94] did not affect error (**Table 2**).

Working memory: In terms of reaction time, we found a significantly decreasing effect of the factor ‘time’ [*F*(1,1) = 16.0, *p* ≤ 0.001], but no significant effect of the factors ‘infusion’ [*F*(1,3) = 0.15, *p* = 0.88] and ‘time x infusion’ [*F*(1,3) = 1.5, *p* = 0.23] on the parameter ‘working memory.’ In terms of error, we found a significantly decreasing effect of the factor ‘time’ (*p* ≤ 0.05), but no significant effect of the factors ‘infusion’ [χ^2^(3) = 5.7, *p* = 0.13] and ‘time x infusion’ [χ^2^(7) = 12.3, *p* = 0.09] on the parameter ‘working memory’ (**Table 2**).

Incompatibility: Reaction time was not affected in terms of the factors ‘time’ [*F*(1,1) = 0.20, *p* = 0.66], ‘infusion’ [*F*(1,3) = 0.24, *p* = 0.81] and ‘time x infusion’ [*F*(1,3) = 2.4, *p* = 0.12]. The factors ‘time’ (*p* = 0.59), ‘infusion’ [χ^2^(3) = 2.2, *p* = 0.54] and ‘time x infusion’ [χ^2^(7) = 3.3, *p* = 0.85] had no significant effect on error (**Table 2**).

#### Olfactory Parameters

**FIGURE 7:**
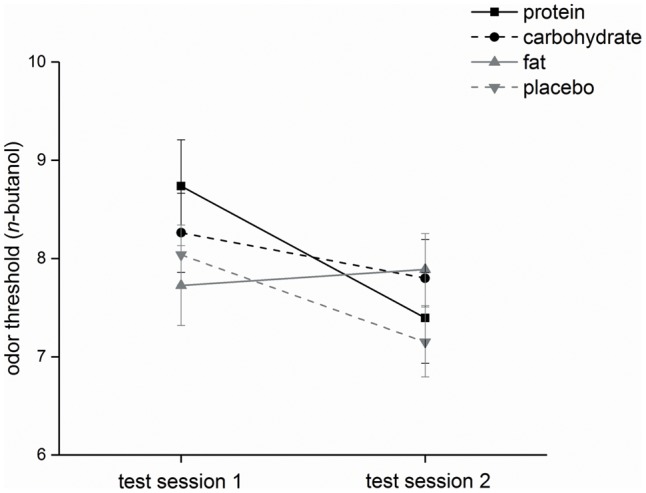
Olfactory parameter (study II). Mean values and standard errors of means of the olfactory parameter odor threshold for all participants (*n* = 20) during intravenous protein, carbohydrate, fat and placebo infusion. Test session 2 took place in the on-infusion status and test session 1 in the pre-infusion status.

Threshold: The factors ‘time’ (*p* ≤ 0.001) and ‘time x infusion’ [χ^2^(7) = 18.1, *p* ≤ 0.05] significantly influenced subjects’ *n*-butanol threshold (**Figure 7**), but ‘infusion’ [χ^2^(3) = 1.5, *p* = 0.67] had no significant influence on the *n*-butanol threshold. *Post hoc* analysis demonstrated that the *n*-butanol threshold was significantly lower at test session 2 compared to test session 1 for protein (*p* ≤ 0.001) and placebo (*p* ≤ 0.01) administration, while administration of carbohydrate (*p* = 0.27) and fat (*p* = 0.78) showed no significant effects. However, the comparison of *n*-butanol threshold at each measurement point showed that there were no significant differences [time 1: χ^2^(3) = 2.6, *p* = 0.47; time 2: χ^2^(3) = 2.6, *p* = 0.46]. The delta of the four intravenous infusions did not significantly differ [χ^2^(3) = 6.3, *p* = 0.097].

Discrimination: The factors ‘time’ [*F*(1,1) = 0.49, *p* = 0.50] and ‘time x infusion’ [*F*(1,3) = 1.1, *p* = 0.06] had no significant influence on discrimination scores, but ‘infusion’ [*F*(1,3) = 3.7, *p* ≤ 0.05] significantly affected discrimination scores. However, *post hoc* analyses did not show significant differences between the four intravenous infusions. The delta of the four intravenous infusions did not significantly differ [*F*(1,3) = 0.88, *p* = 0.45].

Identification: The factors ‘time’ [*F*(1,1) = 0.43, *p* = 0.52], ‘infusion’ [*F*(1,3) = 1.5, *p* = 0.23] and ‘time x infusion’ [*F*(1,3) = 1.3, *p* = 0.29] had no significant effect on subjects’ identification scores. The delta of the four intravenous infusions did not significantly differ [*F*(1,3) = 0.92, *p* = 0.42].

## Discussion

Our studies clearly demonstrated that a mixed nutrient solution of 600 kcal orally consumed within 30 min (volume: 1500 ml) can significantly reduce hunger and food craving. However, this effect disappeared if the ingestion of 600 kcal/1.5 L was spread over a time period of 340 min (low rate of intake, see **Figure 2**). Independent of the type of nutrient solution – protein, carbohydrate, fat, placebo – intravenous infusion also failed to reduce hunger and food craving if the infusion of 600 kcal/1.5 L was spread over a time period of 340 min (low rate application, see **Figure 5**).

Spreading energy and volume over 340 min: Using the Harris-Benedict Equation, the average basal metabolic rate in German men is 1812 kcal/day and 1405 kcal/day in women (Harris and Benedict, 1918; Statistisches Bundesamt, 2011, 2013). Thus, our studies used nutrient solutions and intravenous infusions at a low caloric dimension [isoenergetic (600 kcal)], i.e., near the kcal of one ham- and cheese sandwich (French bread). The low energy per time rate generated by spreading the consumption / infusion of the nutrient solutions over a larger time period can be an explanation of our results. Food form also affects satiety and even spontaneous eating events. Mattes and Campbell (2009) demonstrated that ingestion of solid food led to greater satiation and longer intervals from test food consumption to spontaneous eating events compared to semi-solid and liquid food ingestion.

Another explanation for the lack of effects of low rate applications of 600 kcal could also be the fact that the volume of 1.5 L spread over a time period of 340 min generates a low volume per time rate. This minimizes gastric distension and reduces the activation of the vagal nerve and subsequent neural circuits. In a nasogastric tube feeding study, Stratton et al. (2008) showed that subjects rated their hunger feeling lower directly after bolus application; thereafter the sensation of hunger increased. Short-term continuous nasogastric tube feeding failed to suppress appetite and food intake (Stratton et al., 2003). However, it was not clear whether this failure to suppress appetite and food intake was related to the rate of entry or the route of entry of food. Based on (1), the results by Stratton et al. (2008, 2003) and (2), the results of our studies, it is very likely that the failure to suppress appetite and food intake is related to the rate of intake (energy and volume) and independent of the route of food application, i.e., nasogastric or oral intake.

The slow intervalled rate of oral nutrient intake did not significantly influence hunger and food craving. An explanation of this finding could be the fact that food ingestion that did not reach a critical threshold for gastric distension and/or a critical threshold for energy per time. The effect of ingested volume on satiety was investigated by Rolls et al. (1998). The authors demonstrated that the volume of ingested isoenergetic drinks is an important determinant of satiety. They reported that ingestion of 600 mL, which results in greater stomach distension, led to higher satiety compared to 300 mL and 450 mL. In a further study, Rolls et al. (2000) analyzed the effect of food volume independent of energy density on satiety. The results also confirmed that ingestion of higher volumes leads to higher satiety. Both studies show that the combination of energy and high volume is necessary to feel satiated.

Food craving and hunger: Food craving can be described as wanting to consume food (Martin et al., 2011). Finlayson et al. noted that the incentive-driven process of ‘wanting’ is most likely to be operating on a subcortical mesolimbic level and could occur even in the absence of a cognitive rationale and of conscious awareness (Finlayson et al., 2007). Thus, following the perception of food, craving can also occur in the absence of a cognitive rationale. Functional magnetic resonance imaging studies show centers in the brain that are thought to be involved in food craving: the core of the nucleus accumbens, the broader ventral striatum, basolateral amygdala and ventral tegmental area (Berridge, 2009; Volkow et al., 2011). However, the estimation of (macro)nutrient content may present a cognitive task that is associated with intelligence and educated intellectual abilities. In our studies, we were interested in the intuitive assessment of food craving concerning different types of food. In our experiments in study I, the rate of intake showed a significant influence on food craving over time, i.e., following normal rate of intake, food craving significantly decreased for all types of food craving measured. Regarding study II, none of the 600 kcal intravenous nutrient infusions (fat or protein or carbohydrates) or placebo, applied intravenously changed the perception of any type of food craving if applied over this long time period. Hill and Heaton-Brown (1994) reported that food craving can be characterized as a hunger-modifying, mood-improving experience that is directed at wanting to consume highly pleasant-tasting food. In another study, Hill et al. (1991) demonstrated that food craving is very often associated with hunger. Our results support these findings because hunger and craving sensations showed similar patterns over time.

Cognitive, olfactory and metabolic effects: Regarding study I, we only found (1) a significant reduction of error of the more complex cognitive task ‘incompatibility’ for test session 2 during normal rate of intake, (2) a significant increase in odor identification scores for test session 2 during slow intervalled rate of intake, and (3) higher intensity ratings at test session 2 independent of rate of intake. Hunger and craving estimates did not differ at the beginning of test session 2 (240 min after the beginning of the intake, see **Figures 1, 2**). Thus, the differences in incompatibility error and odor identification observed in study I are related to the rate of intake only. Regarding study II, we only found that administration of an intravenous fat infusion improved threshold scores at test session 2 compared to test session 1, while administration of the other intravenous infusions showed contrary results, with significant effects for protein and placebo administration (see **Figure 7**). Based on our studies we cannot decide if the improvement observed is a central or peripheral effect, e.g., related to changes in cell membrane of olfactory sensory neurons by fatty acids. Researchers also observed an improvement of olfactory sensitivity in the satiated state compared to the hunger state using different olfactory tests (Albrecht et al., 2009; Stafford and Welbeck, 2011). In contrast, Guild (1956) demonstrated that olfactory sensitivity was highest before and least after satisfying meals. However, these studies investigated oral food administration, while our study II bypassed the gastrointestinal system. For the slow intervalled rate of intake, we observed an increase in blood plasma glucose level followed by a slow decrease in glucose level as described in the literature (McCarthy et al., 1977). This time course can be easily explained by the low insulin release elicited by the small ingested amounts during the slow intervalled rate of intake condition. In contrast, the ingestion of the 600 kcal during normal rate of intake leads to a high and effective insulin release, eliciting a high decrease in glucose level. The most likely explanation for the high insulin release following normal rate of oral intake is a synergistic effect of the protein and fat ingredients and glucose (Gannon et al., 1992; van Loon et al., 2000, 2003; Itoh et al., 2003; Frid et al., 2005).

In our studies we could show different effects of the consumption of macronutrients on hunger, food craving and metabolic parameters in relation to energy per time rate and volume per time rate. The influence of the volume per time rate also motivates to investigate the modulation of vagal afferent input in dietary research. Previous studies have shown that the TRPV1 agonist capsaicin can acutely sensitize humans to gastric distension (Lee et al., 2004) and reduces food intake (Yoshioka et al., 1999). A recent study in mice demonstrated that TRPV1 channels modulate gastric vagal afferent tension receptor mechanosensitivity (Kentish et al., 2015). The researchers suggested that the reduced activity of gastric vagal afferents to distension in a high fat diet condition is due to disruption of TRPV1 channel signaling. In TRPV1^-/-^ mice no reduction was observed in gastric tension receptor mechanosensitivity for the condition of high fat diet-induced obesity. Based on the assumption that gastric distension mediates – at least in part – the effects observed in our study I, further research of the modulating effects of TRPV1 agonists on gastric vagal afferents is desirable.

Analyzing the area under the curve of hunger and craving ratings over a given time period could be an effective method to improve dietary therapies or artificial feeding. Based on the results of our studies further research should concentrate on effective energy and volume per time rates in order to reduce the area under the curve of the ratings of hunger and food craving. However, it is also important to extend the view to other parameters and behavioral aspects. Bolhuis et al. (2013), e.g., demonstrated that consuming soup with larger sips results in higher food intake compared to small sips. In addition, it has been shown that prolonged orosensory exposure of food can help to reduce food intake (Bolhuis et al., 2011).

Limitations of our pilot studies are the relatively small number of subjects and the fact that only male participants were included. To confirm our preliminary results, further studies with higher numbers of male and female participants are requested. Beyond the scope of food craving, hunger and satiety, future studies should also investigate the effects of different oral intake rates at a behavioral level, e.g., on subsequent food consumption.

## Conclusion

Normal oral intake significantly reduces hunger and food craving compared to isocaloric slow intervalled oral intake and intravenous low rate macronutrient application. This implies that there is a threshold rate for suppressing hunger and food craving which is probably related to the volume of the food ingested and the energy per time ratio. Thus, specific studies determining the threshold rates of gastric distension and energy per time are requested in order to improve the management of hunger and food craving during artificial feeding and during oral diets.

## Author Contributions

Each author has participated sufficiently in the work, intellectually or practically, to take public responsibility for the content of this article, including the conception, design, and conduct of the experiment and data analysis and interpretation. MD-L, SB, and JW were responsible for subject recruiting. MD-L and SB carried out the practical work. MD-L and SB were responsible for data analysis. AB, AD, CS, JK, and NT conceived the study, and MD-L, SB, JW, and MF participated in the design of the study. All authors contributed to the manuscript and approved the final version.

## Conflict of Interest Statement

The authors declare that the research was conducted in the absence of any commercial or financial relationships that could be construed as a potential conflict of interest.

## Abbreviations

DSM-IV: diagnostic and statistical manual of mental disorders-IV
FEV: Fragebogen zum Essverhalten
